# Optimized Whole-Genome Amplification Strategy for Extremely AT-Biased Template

**DOI:** 10.1093/dnares/dsu028

**Published:** 2014-09-19

**Authors:** Samuel O. Oyola, Magnus Manske, Susana Campino, Antoine Claessens, William L. Hamilton, Mihir Kekre, Eleanor Drury, Daniel Mead, Yong Gu, Alistair Miles, Bronwyn MacInnis, Chris Newbold, Matthew Berriman, Dominic P. Kwiatkowski

**Affiliations:** 1 Wellcome Trust Sanger Institute, Hinxton, UK; 2 MRC Centre for Genomics and Global Health, University of Oxford, Oxford OX3 7BN, UK; 3 Wellcome Trust Centre for Human Genetics, University of Oxford, Oxford OX3 7BN, UK; 4 Weatherall Institute of Molecular Medicine, University of Oxford, Oxford OX3 9DS, UK

**Keywords:** whole-genome amplification, AT-rich, malaria, tetramethylammonium chloride

## Abstract

Pathogen genome sequencing directly from clinical samples is quickly gaining importance in genetic and medical research studies. However, low DNA yield from blood-borne pathogens is often a limiting factor. The problem worsens in extremely base-biased genomes such as the AT-rich *Plasmodium falciparum*. We present a strategy for whole-genome amplification (WGA) of low-yield samples from *P. falciparum* prior to short-read sequencing. We have developed WGA conditions that incorporate tetramethylammonium chloride for improved amplification and coverage of AT-rich regions of the genome. We show that this method reduces amplification bias and chimera formation. Our data show that this method is suitable for as low as 10 pg input DNA, and offers the possibility of sequencing the parasite genome from small blood samples.

## 1. Introduction

Timely detection of emerging genetic variants and other evolutionary features associated with important clinical phenotypes such as increased virulence and drug resistance are central to malaria control strategies. Genome sequencing of parasite populations has been identified as an effective tool for detecting genetic changes.^1,2^ Despite the current success in the sequencing technology, there remain significant challenges in achieving global genetic surveillance of parasite populations in the field. Most genome-scale analyses, such as whole-genome sequencing, require large amounts of clean genetic material that is often difficult to obtain,^3^ and therefore a serious impediment to genetic analysis on many clinical samples. A large number of valuable clinical specimens are collected in the form of small samples that yield low quantity and quality of genetic material.^4–7^ A common method for collecting clinical samples in the field is through heel/finger-pricks.^5,7–10^ However, the quantity and quality of parasite genetic material that can be extracted from these small blood samples usually fall below the threshold required by genome sequencing platforms.

To alleviate the problem of low DNA quantities, whole-genome amplification (WGA) is now routinely applied in many applications,^3,11^ but has yet to be optimized for use in genomes of extreme base composition such as *Plasmodium falciparum*. Two major forms of WGA have been described: multiple displacement amplification (MDA)^12,13^ and PCR-based amplification methods.^14,15^ MDA has been the method of choice for a wider range of genome amplification studies, because it produces longer DNA products with extensive genome coverage.^16^

MDA is based on φ29 polymerase, which, in the presence of random hexamers annealed to denatured DNA, uses an MDA mechanism to synthesize high-molecular-weight DNA from very minute amounts of input material under isothermal conditions.^17,18^ The best results, however, have been obtained from genomes with relatively balanced base composition.^11,19,20^ Amplification of genomes with imbalanced base composition, such as the AT-rich *P. falciparum*, has remained a challenge.^21,22^

In this study, we sought to identify and optimize a WGA system suitable for an AT-base-biased genome of *P. falciparum*. Using standard conditions as outlined for each system, we tested the efficiency of non-MDA- and MDA-based methods. Initial findings showed that MDA-based systems produced a more uniform genome coverage than non-MDA methods (data not shown). We have optimized an identified MDA system to produce an improved genome coverage and a reduced base-bias with more accurate genome representation. We show that our optimized WGA conditions are suitable for as low as 10 picograms (pg) *P. falciparum* input DNA, producing high-sequence concordance with unamplified genomic DNA. This development promises a significant tool to aid implementation of the global genetic surveillance of parasite populations through small blood sample sequencing.

## 2. Materials and methods

### 2.1. DNA samples

*Plasmodium falciparum* 3D7 genomic DNA was a gift from Prof. Chris Newbold (University of Oxford). The clinical isolates were obtained from the Malaria Genetics Group's Sequencing Sample Repository at the Wellcome Trust Sanger Institute. Other genomic DNA was extracted from 17 progeny clones of *P. falciparum* strains derived from genetic cross between 7G8xGB4^23^ and a 3D7 strain (3D7_glasgow).

### 2.2. Whole-genome amplification

All non-MDA WGA were performed following individual kit manufacturer's instructions. MDA-based WGA was performed using either REPLI-g Mini kit (Qiagen) or Genomiphi kit (GE Healthcare). For Genomiphi, the kit manufacturer's instructions were followed without modification. For the REPLI-g Mini kit, manufacturer's instructions were followed during preliminary tests. The following modifications were performed in developing optimized conditions for the REPLI-g Mini kit: nuclease-free water and all tubes were UV-treated before use. WGA reactions were performed in 0.2 ml PCR tubes. Buffer D1 stock solution (Qiagen) was reconstituted by adding 500 µl of nuclease-free water, and a working solution was prepared by mixing the stock solution and nuclease-free water in the ratio of 1 : 3.5, respectively. Unmodified Buffer N1 was reconstituted by mixing Stop solution (Qiagen) and nuclease-free water in the ratio of 1 : 5.7. Modified buffer N1 was prepared by including tetramethylammonium chloride (TMAC) at a concentration of 300 mM. To denature DNA templates, 5 µl of the DNA solution was mixed with 5 µl of buffer D1 (working solution prepared as described above). The mixture was vortexed and centrifuged briefly before incubating at room temperature for 3 min. Denatured DNA was neutralized by adding 10 µl of either unmodified or modified buffer N1. Neutralized DNA was mixed by vortexing and centrifuged briefly. To amplify the DNA template, denatured and neutralized sample was mixed with 29 µl of REPLI-g Mini Reaction Buffer and 1 µl of REPLI-g Mini DNA polymerase to obtain a final reaction volume of 50 µl. The reaction mixture was incubated at 30°C for 16 h using an MJ Research PTC-225 thermal cycling system (GMI, Inc., USA) with the heating lid set to track at +5°C. Amplified DNA was cleaned using Agencourt Ampure XP beads (Beckman Coulter) using sample to beads ratio of 1 : 1 and eluted with 50 µl of EB (Qiagen).

### 2.3. Illumina library preparation and sequencing

All sequencing libraries were prepared as PCR-free. Whole-genome amplified or unamplified genomic DNA (1.5 μg in 75 µl of TE buffer) was sheared using a Covaris S2 (Covaris, Inc., Woburn, MA, USA) to obtain a fragment-size distribution of ∼300 to ∼600 bp. The sheared DNA fragments were end-repaired and A-tailed using the NEBNext DNA sample preparation kit (NEB), following an Illumina sample preparation protocol. Pre-annealed paired-end Illumina PCR-free adapters were ligated to the A-tailed fragments in a 50-µl reaction containing 10 µl of DNA sample, 1× Quick T4 DNA ligase buffer, 10 µl of PCR-free PE-adapter mixture, 5 µl of Quick T4 DNA ligase (NEB) and incubated at 20°C for 30 min. The ligation reaction was cleaned twice using Agencourt Ampure XP beads (Beckman Coulter). Cleaned DNA was eluted with 20 µl of buffer EB. Aliquots were analysed using an Agilent 2100 Bioanalyzer (Agilent Technologies) to determine the size distribution and to check for adapter contamination. Samples were sequenced using either Illumina Hiseq 2500 or Miseq technologies (San Diego, CA, USA) with 75 bp read length and the paired-end read options. Corresponding WGA and non-WGA samples were run in the same lanes, using different multiplex tags. This strategy reduces potential confounding artefacts relating to sequencing chemistry.

### 2.4. Read mapping and genotype concordance analysis

Reads were mapped against the *P. falciparum* reference sequence (http://plasmodb.org/common/downloads/release-10.0/Pfalciparum3D7/fasta/data/), using BWA (V0.6.2). We analysed the genotype calls from 17 samples derived from the progeny clones of genetic crosses of 7G8xGB4 laboratory strains,^23^ including one parent reference strain 3D7 as a control. For genotype concordance analysis, we generated *de novo* variation calls using samtools (V0.1.1.19) mpileup and bfctools (V0.1.17), calling on both WGA and non-WGA samples. For *in silico* genotyping, we used a list of 20 737 high-quality single-nucleotide polymorphism (SNP) positions and alleles generated from the sequence of genetic crosses (unpublished data). We performed *in silico* genotyping of both the WGA and non-WGA samples using samtools mpileup and counting alleles present in at least five reads.

## 3. Results

### 3.1. WGA using standard methods

**Figure 1.**
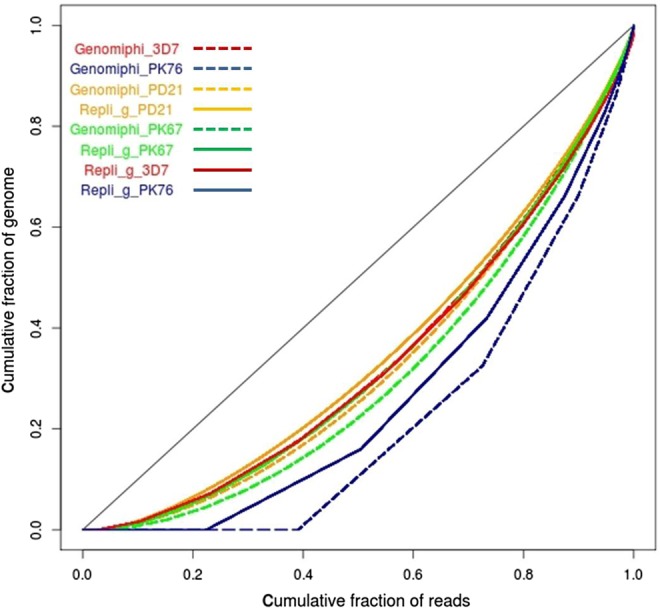
Lorenz curves of genome coverage analysis. Cumulative fraction of sequence reads against cumulative fraction of genome covered is shown for each sample. The diagonal line represents an ideal perfect uniform coverage. The further the sample curves deviate from the diagonal line the more bias in genome coverage they are. Repli_g (solid lines) and Genomiphi (dashed lines) sample pairs were normalized to equal number of reads. Repli_g and Genomiphi amplification of *P. falciparum* 3D7 genomic DNA (3D7) resulted in identical coverage uniformity as shown in overlapping solid red (Repli_g_3D7) and dashed red (Genomiphi_3D7) curves, respectively. Amplification of clinical samples with varied proportions of human DNA contaminations produced different uniformity of coverage with Genomiphi (dashed lines) showing slightly more bias than their corresponding Repli_g (solid lines) amplified samples. 3D7, pure *P. falciparum* genomic DNA; PK76, clinical sample with 53% host DNA contamination amplified with either Genomiphi (Genomiphi_PK76) or Repli_g (Regpli_g_PK76); PD21, a clinical sample with 7% host contamination amplified with either Genomiphi (Genomiphi_PD21) or Repli_g (Regpli_g_PD21); PK67, a clinical sample with 19% host contamination amplified with either Genomiphi (Genomiphi_PK67) or Repli_g (Regpli_g_PK67).

We performed tests on various WGA systems to identify those suitable for AT-rich *P. falciparum* genome. We compared non-MDA—Endcore-Rapid (NuGen), MALBAC (Yikon Genomics), Rapisome (Biohelix) and MDA—Repli_g (Qiagen) and Genomiphi (GE Healthcare) systems. Our preliminary data (not shown) indicated that non-MDA methods were less tolerant to the AT-biased genome compared with their MDA counterparts. With non-MDA amplification, we observed excess bias towards regions with high GC content and poor coverage of high AT regions of the genome resulting into high levels of allele dropout. Further optimization focused on the MDA systems. Repli_g and Genomiphi are the most commonly used methods for MDA. Although both kits have shown similar results in many systems,^24,25^ the majority of studies have been with genomes of relatively balanced base composition. Here, we have assessed the performance of Repli_g and Genomiphi by amplifying an AT-rich *P. falciparum* genome. Amplification of pure *P. falciparum* 3D7 genomic DNA by both kits produced very similar results as assessed by genome coverage analysis metrics. However, amplification of host-contaminated clinical samples produced dissimilar results in terms of base composition and overall genome coverage (Fig. 1). Whereas amplification products generated by both Repli_g and Genomiphi did not maintain the exact original host/parasite DNA proportions, Genomiphi products were more biased towards the host DNA (Fig. 1), an observation suggesting bias towards templates of neutral base composition originating from the host genome. Based on the level of bias, we chose to optimize Repli_g for amplification of the *P. falciparum* genome.

### 3.2. Optimized WGA conditions reduce amplification bias and improve coverage on low complexity regions

**Figure 2.**
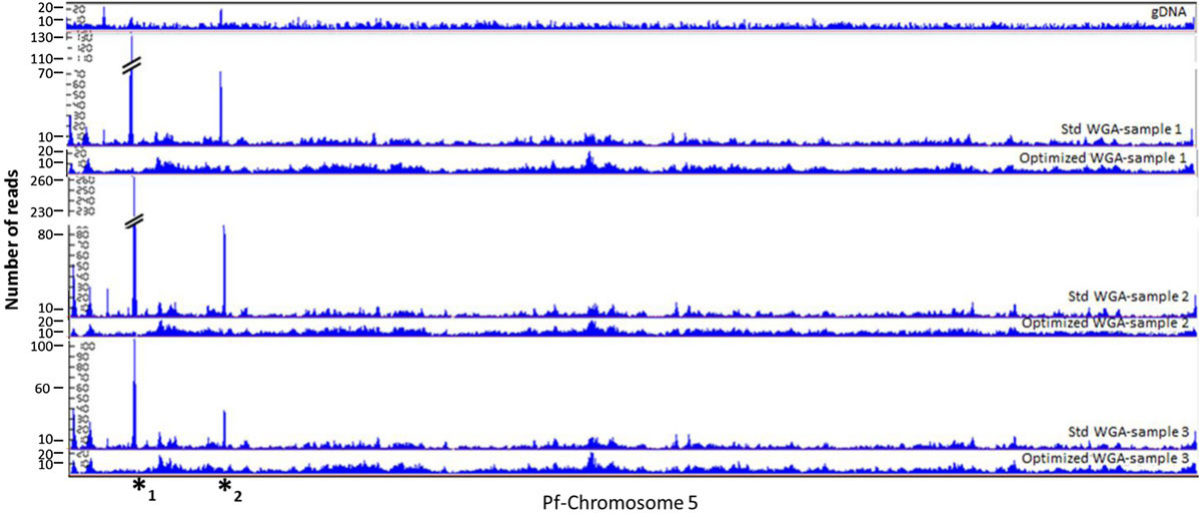
LookSeq analysis of coverage uniformity. *Plasmodium falciparum* 3D7 genomic DNA amplified with Repli_g following standard or optimized procedures. gDNA (top panel) shows bulk genomic DNA sequenced without amplification. All samples amplified using the standard (Std WGA) procedure show regions of amplification bias (marked by *1 and *2, see Supplementary Table S1), whereas their corresponding counterparts amplified following the optimized procedure (Optimized WGA) show less bias and relatively uniform coverage. Different amounts of input DNA were used for each sample set (Sample 1, 0.1 ng; Sample 2, 0.5 ng; Sample 3, 1 ng).

**Figure 3.**
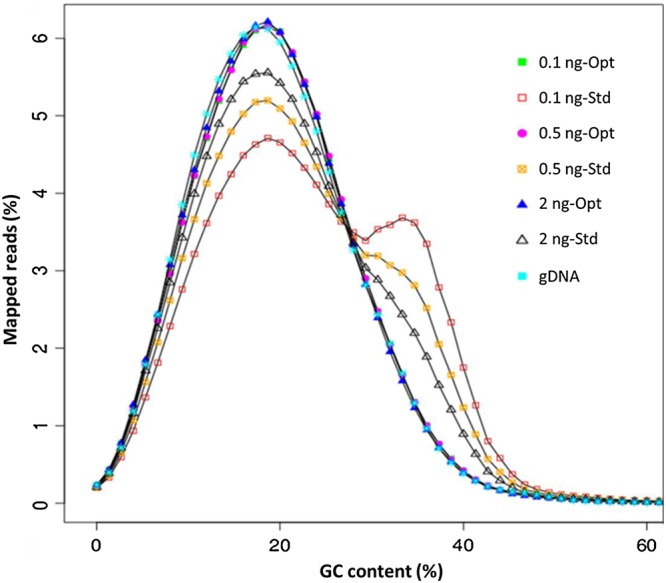
GC content analysis. Different amounts of *P. falciparum* 3D7 genomic DNA (ranging from 0.1 to 2 ng as shown in sample names) were used as an input template for amplification by Repli_g following the standard or optimized procedure. Amplification products were sequenced as PCR-free and reads obtained were analysed for G+C content profile. A non-WGA sample (gDNA) shows a GC content of ∼19.4%, a profile that was closely matched by products amplified using the optimized procedure (Opt). Samples amplified following the standard procedure (Std) showed biased GC content shown as a shift towards the neutral base composition.

Non-coding regions of *P. falciparum* DNA contain ∼90% A+T base composition. Amplification of AT-rich genomes is a challenge to almost all commercially available polymerases. We have previously shown that addition of TMAC improves coverage of low GC regions of the genome during PCR,^22^ but the same has not been tested with φ29, the MDA polymerase. To investigate the effect of TMAC on MDA, we amplified *P. falciparum* 3D7 genomic DNA of varied input amounts (0.1–2 ng) both in the presence and absence of TMAC. We compared the quantity and quality of both products and observed that, like many commercial PCR polymerases, φ29 is inhibited by TMAC at certain levels of concentration. We have determined 60 mM as a concentration that is non-inhibitory to the polymerase and optimal for WGA of an AT-biased genome. Although the quantity of the amplification product was higher in the absence (standard procedure) than in the presence of 60 mM TMAC (data not shown), the quality of MDA product, in terms of coverage and base composition, was improved in the optimized procedure where TMAC was added (Figs 2 and 3). As shown in Fig. 2, amplification using a standard protocol (Std) resulted in excessive bias in regions of low complexity. Inspection of the over-amplified regions reveals sequences of low complexity and numerous repeat patterns. We used a tandem repeat finder programme^26^ and revealed numerous sequences in tandem repeat conformation that may have affected amplification bias. The top three tandem repeats are provided in Supplementary Table S1. Unlike the standard WGA protocol, our optimized amplification procedure abolished excessive amplification bias and produced a more uniform coverage. The mechanism by which this bias is corrected is not clear, but it is conceivable that TMAC stabilizes and stiffens the DNA backbone, thereby minimizing *cis*-priming by the looping of displaced DNA strands during amplification. We also show that base composition bias increased with a decrease in the amount of input DNA during amplification with standard protocols. This bias was not observed in samples amplified with the optimized (Opt) conditions (Fig. 3).

### 3.3. Optimized conditions reduce chimera formation and maintain template base composition during MDA by φ29

**Figure 4.**
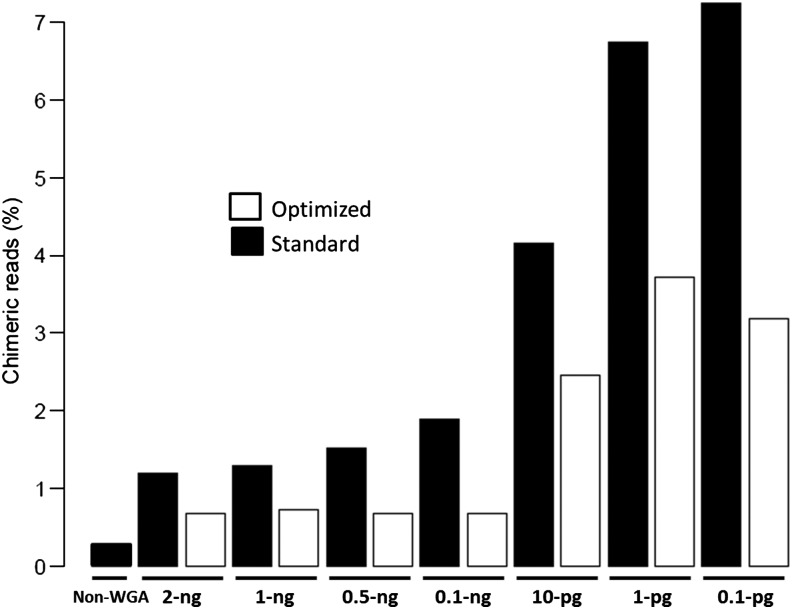
Bar graph of chimeric read analysis. Different amounts of *P. falciparum* 3D7 genomic DNA (ranging from 2 ng to 100 fg as shown in sample labels) were used as an input template for amplification by Repli_g following the standard or optimized procedure. Amplification products were sequenced as PCR-free, and reads obtained were normalized and analysed for the presence of chimeric reads. A non-WGA sample (control) showed the least number of chimers. Using the standard procedure, the number of chimers increased with a decrease in the amount of input DNA. Samples amplified using the optimized procedure showed a decrease in chimer formation that remained low and steady from 2 ng to 100 pg of input DNA.

Formation of chimeric reads is a major problem with MDA technology.^27^ Chimeras cause serious mapping and assembly problems, and therefore reduce the quality and quantity of the WGA product. We analysed the formation of chimeras by comparing WGA products following standard and optimized procedures. The number of chimeric reads increased as the amount of input DNA was reduced (Fig. 4). However, optimizing WGA condition by including TMAC additive decreased the formation of chimeras and improved the quality of reads in *P. falciparum* WGA.

### 3.4. WGA from 10 pg of input P. falciparum genomic DNA

**Figure 5.**
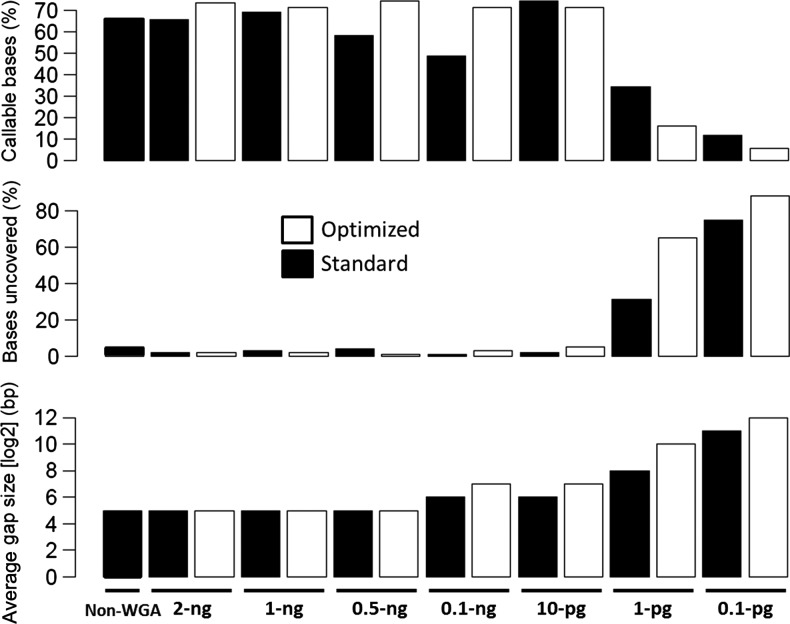
Determining the lowest threshold of input DNA mass for WGA. Different amounts of *P. falciparum* 3D7 genomic DNA (ranging from 2 ng to 100 fg, as shown in sample names) were used as an input template for amplification by Repli_g following the standard or optimized procedure. Samples were multiplexed and sequenced using a fast turn-around Illumina Miseq machine. Sequence reads mapped to the reference were normalized and analysed for coverage and base quality using the ‘CallableLoci’ program of GATK. A non-WGA sample was used as an unamplified control. Input quantities ranging from 2 ng down to 10 pg produced reads with high-quality ‘callable’ bases covering over 60% of the genome. Input DNA below the10-pg threshold produced poor base quality with only <30% of genome covered with ‘callable’ bases. Input DNA <10 pg showed a sharp increase in positions with zero coverage (bases uncovered) and an increase in gap sizes in genome coverage.

Most studies with φ29 MDA have used an input DNA of ≥10 ng for WGA.^19,20^ In many cases, this amount may be difficult to obtain from valuable clinical specimens. We set to find out the minimal amount of parasite DNA that can be successfully amplified by the optimized conditions to obtain uniform genome coverage. We performed WGA on *P. falciparum* genomic DNA with an input amount ranging from 2 ng down to 100 femtograms (fg). WGA products were multiplexed and sequenced using Illumina MiSeq or HiSeq 2500 machines. Sequence reads generated were analysed to determine the minimum threshold required to produce optimal coverage suitable for various whole-genome studies including genotyping by SNP analysis. To assess the quality of the sequence data generated from each input amount, reads were mapped to the reference genome using BWA. CallableLoci program of the genome analyser tool kit (GATK)^28^ was used to inspect and count the proportion of the genome with high-quality base coverage (callable bases), positions of the genome with zero coverage (uncovered bases) and the size of coverage gaps. Using these metrics, we show that the number of callable loci (high-quality bases) remained relatively high for samples with input DNA ranging from 2 ng to 10 pg. However, the quality of sequence data dropped sharply for samples with input DNA <10 pg (Fig. 5). A similar trend was observed with the proportion of gap sizes (length of uncovered bases, Fig. 5 bottom panel) and chimeric reads (Fig. 4), where a sharp increase in these values was observed for samples with input DNA <10 pg. From these observations, we concluded that 10 pg is the minimal amount of *P. falciparum* input DNA that produces quality genome coverage under the optimized MDA conditions described. This amount of DNA is equivalent to only ∼380 parasite genomes.

### 3.5. Detailed analysis of WGA products from a 10-pg input template DNA

Standard WGA methods produce products that show sequence representation bias, allelic dropout and amplification artefacts. These problems tend to increase as the amount of input template decreases. To adequately evaluate the quality of the WGA products produced from an input of 10 pg—1,000-fold smaller than the standard input amounts—we performed analysis on genomic DNA extracted from *P. falciparum* strains derived from the progeny clones of genetic cross between 7G8xGB4 laboratory strains, as well as the 3D7 Strain. Genomic DNA extracted from 17 progeny clones was sequenced both as WGA and as bulk genomic DNA (non-WGA). We performed detailed analysis by comparing sequence data generated from WGA and their matching unamplified genomic DNA (non-WGA). WGA and non-WGA samples yielded a median of 3.5 and 3.4 billion base sequences, respectively, with 94.8 and 95.3% of the read mapping to the reference sequence (Table 1). WGA samples showed coverage between 180× and 500×, whereas non-WGA samples showed coverage between 90× and 250×. A median of 1.5 and 1.2% of genome bases was not covered in WGA and non-WGA samples, respectively (Table 1).

**Table 1.** Sequence coverage and mapping analysis

Sample	Total reads (million)		Total base (Gb)			Mapped reads (%)		Uncovered bases (%)	
	WGA	Non-WGA	WGA	Non-WGA	WGA/non-WGA	WGA	Non-WGA	WGA	Non-WGA
3D7_Glasgow	30.8	43.5	2.3	3.3	0.7	98.8	98.6	0.2	0.0
DEV_18_05_11	74.5	50.7	5.6	3.8	1.5	94.6	95.0	1.1	1.2
D2_18_05_11	41.7	41.4	3.1	3.1	1.0	94.2	95.2	1.2	1.0
WE2	69.7	35.2	5.2	2.6	2.0	95.1	95.1	1.2	1.3
GB4_NIH	45.2	37.9	3.4	2.8	1.2	94.2	94.5	1.3	1.1
JC3	45.1	47.6	3.4	3.6	0.9	94.3	95.1	1.3	1.1
JF6	49.1	57.3	3.7	4.3	0.9	94.3	95.5	1.4	1.1
QF5	46.5	66.6	3.5	5.0	0.7	95.2	95.9	1.4	1.2
NIC_18_05_11	59.1	36.9	4.4	2.8	1.6	95.5	90.6	1.5	1.3
NF10	48.4	35.0	3.6	2.6	1.4	95.5	95.0	1.5	1.4
XF12_18_05_11	39.2	58.6	2.9	4.4	0.7	94.7	95.6	1.5	1.2
AL2_13_05_11	38.3	40.1	2.9	3.0	1.0	94.8	95.4	1.5	1.3
XD8	46.9	45.0	3.5	3.4	1.0	93.6	95.5	1.6	1.2
7G8_NIH	61.1	40.5	4.6	3.0	1.5	94.2	94.9	1.6	1.5
JON	51.3	71.8	3.8	5.4	0.7	94.0	95.4	1.6	1.1
TF1	57.7	65.2	4.3	4.9	0.9	95.5	95.7	1.8	1.4
JC9	34.0	61.3	2.5	4.6	0.6	95.0	95.6	2.5	1.2
Median	46.9	45.0	3.5	3.4	1.0	94.7	95.4	1.5	1.2
Mean ± SD	49.3 ± 12	49.1 ± 12	3.7 ± 0.9	3.7 ± 0.9	1.0 ± 0.4	94.9 ± 1.2	95.2 ± 1.5	1.4 ± 0.4	1.1 ± 0.3

Sequence reads obtained from WGA and non-WGA samples were analysed for coverage and mapping statistics. Nearly identical average number of sequence reads, mapping and coverage statistics were obtained for both WGA and non-WGA datasets. However, coverage distribution was less uniform in WGA compared with non-WGA datasets.

### 3.6. Genotype concordance analysis

To evaluate the fidelity of sequence representation by WGA, we analysed the genotype calls from 17 cross samples and the parent reference strain. We performed *de novo* and *in silico* genotype calls on sequence generated from both the WGA and non-WGA samples. Genotype concordance was determined by comparing SNP and InDel calls from matched pairs of both non-WGA and WGA samples.

#### 3.6.1. Concordance of de novo SNPs and InDel calls

For each pair of WGA and non-WGA sample, we generated *de novo* variation calls simultaneously. A median of 20,338 biallelic *de novo* SNPs with a quality score of ≥250 were called for each sample. For the 3D7 reference strain (3D7_Glasgow), only 185 *de novo* SNPs were called (Supplementary Table S2). Both of these numbers are well within the expected range for this method.^1^ As given in Table 2, call pairs were grouped into ‘Perfect Concordance’, ‘WGA New Alleles’ and ‘Undetermined’. Identical calls in both non-WGA and WGA samples had a median of 97.3% and a mean of 96.7% ± 1.2 that are in perfect concordance, and new alleles present only in the WGA samples (considered as WGA-introduced alleles) had a median of 1.2% and a mean of 1.8 ± 1.1%. Most of the WGA-introduced alleles were due to a mixed call in WGA, where the non-WGA counterpart only showed the reference allele. Discordant single-allele calls were extremely rare with a median of zero SNPs and a total of 10 occurrences across all the samples (Supplementary Table S2a). Additionally, *de novo* SNP calls in 5,075,789 well-covered coding positions (Table 2) produced perfect concordance with a median of 98.5% and a mean of 98.0 ± 1.4%, while the occurrence of WGA-introduced alleles dropped to a median of 0.7% and a mean of 0.8 ± 0.4%. In this subset, no discordant calls (alt/ref or ref/alt) were observed (Supplementary Table S2b). Calls that were grouped as ‘Undetermined’ represent alleles that were missing or wrong alleles (present in the non-WGA samples but are neither the reference nor the alternative allele).

**Table 2.** WGA and non-WGA SNP concordance analysis

Sample	*De novo* SNP calls in all regions			*De novo* SNP calls in good quality regions		
	Perfect concordance (%)	WGA new allele (%)	Undetermined (%)	Perfect concordance (%)	WGA new allele (%)	Undetermined (%)
JON	93.8	4.4	1.8	97.2	1.6	1.2
JF6	94.4	4.3	1.3	94.2	5.0	0.8
D2_18_05_11	95.0	2.6	2.4	96.9	2.2	0.9
QF5	95.7	2.5	1.8	99.0	0.5	0.5
QF5	96.3	2.6	1.1	98.5	0.8	0.7
JC9	96.5	1.6	1.9	98.2	1.3	0.5
XF12_18_05_11	97.3	1.4	1.4	98.4	0.3	1.2
NIC_18_05_11	97.3	0.8	2.0	95.6	2.4	2.0
NF10	97.3	1.5	1.3	98.7	0.5	0.7
DEV_18_05_11	97.3	1.1	1.6	98.9	0.5	0.5
JC3	97.4	1.1	1.6	98.6	0.6	0.8
GB4_NIH	97.4	1.1	1.5	98.5	0.5	0.9
AL2_13_05_11	97.4	1.2	1.4	99.0	0.6	0.5
WE2	97.5	1.0	1.6	97.9	1.6	0.5
7G8_NIH	97.5	1.1	1.4	97.1	2.4	0.5
TF1	97.6	1.0	1.3	98.8	0.5	0.7
3D7_Glasgow	97.8	1.1	1.1	100.0	0.0	0.0
Median	97.3	1.2	1.5	98.5	0.6	0.7
Mean ± SD	96.7 ± 1.2	1.8 ± 1.1	1.6 ± 0.3	98.0 ± 1.4	1.3 ± 1.2	0.8 ± 0.4

WGA and non-WGA data were analysed for *de novo* SNP calling concordance. Matching sequence data from cross samples were paired and the SNP calling was performed simultaneously. Concordance rates were generated by comparing WGA and related non-WGA calls. Columns on the left show comparison of all calls covering the entire genome, whereas columns on the right show a subset of all calls covering only the high-quality coding regions of the genome. Values in the perfect concordance columns represent the proportion of calls that were identical in both WGA and non-WGA samples. WGA new allele columns show the proportion of SNPs that were present only in the WGA and not in their matching non-WGA alleles. Undetermined column shows the proportion of SNPs that were called in wrong or missing alleles (Supplementary Table S2).

As with SNPs, we also performed biallelic *de novo* InDel calls with a quality score of ≥250 for both WGA and non-WGA samples. As given in Table 3, InDel calls from the whole genome with perfect concordance between WGA and non-WGA samples had a median of 97.6% and a mean of 96.8 ± 1.7%, and WGA-introduced InDels had a median of 1.5% and a mean of 2.4 ± 1.8%. Similarly, *de novo* InDel calls in the coding regions only produced near-perfect concordance with a median of 98.7% and a mean of 98.2 ± 1.4%. WGA-introduced InDels in these regions dropped to a median of 0.4% and a mean of 1.4 ± 1.3%.

**Table 3.** WGA and non-WGA InDel concordance analysis

Sample	*De novo* InDel calls in whole genome			*De novo* InDel calls in good quality regions		
	Perfect concordance (%)	WGA New InDels (%)	Undetermined	Perfect concordance (%)	WGA New InDels (%)	Undetermined (%)
3D7_Glasgow	95.2	4.8	0.0	100.0	0.0	0.0
JC3	97.6	1.5	0.9	98.8	0.8	0.4
QF5	97.2	2.1	0.6	96.1	3.6	0.3
JF6	91.5	7.8	0.7	96.5	3.4	0.2
TF1	98.2	1.2	0.5	99.4	0.5	0.1
DEV_18_05_11	98.1	1.0	0.8	99.2	0.3	0.5
7G8_NIH	97.9	1.2	0.8	98.7	0.6	0.8
GB4_NIH	97.9	1.2	0.9	98.6	0.7	0.7
WE2	98.0	1.1	0.9	99.0	0.7	0.3
D2_18_05_11	95.8	2.5	1.7	97.1	1.6	1.3
NIC_18_05_11	97.8	1.0	1.2	99.1	0.3	0.6
NF10	97.4	1.8	0.8	98.3	1.1	0.6
XF12_18_05_11	98.0	1.4	0.6	99.0	0.6	0.4
AL2_13_05_11	97.7	1.5	0.8	99.3	0.3	0.3
XD8	95.5	3.9	0.7	95.7	3.6	0.7
JON	95.2	4.2	0.6	96.1	3.7	0.3
JC9	96.4	2.9	0.7	98.4	1.5	0.1
Median	97.6	1.5	0.8	98.7	0.7	0.4
Mean ± SD	96.8 ± 1.7	2.4 ± 1.8	0.8 ± 0.3	98.2 ± 1.4	1.4 ± 1.3	0.4 ± 0.3

WGA and non-WGA data were analysed for *de novo* InDel calling concordance. Calls were performed simultaneously on both WGA and related non-WGA. Columns on the left show comparison of all calls covering the entire genome, whereas columns on the right show a subset of all calls covering only the high-quality coding regions of the genome. Values in the perfect concordance columns represent the proportion of calls that were identical in both WGA and non-WGA samples. WGA new allele columns show the proportion of InDels that were present only in the WGA and not in their matching non-WGA alleles. Undetermined column shows the proportion of InDels that were called in wrong or missing alleles (Supplementary Table S3).

#### 3.6.2. Concordance of in silico genotyping

**Figure 6.**
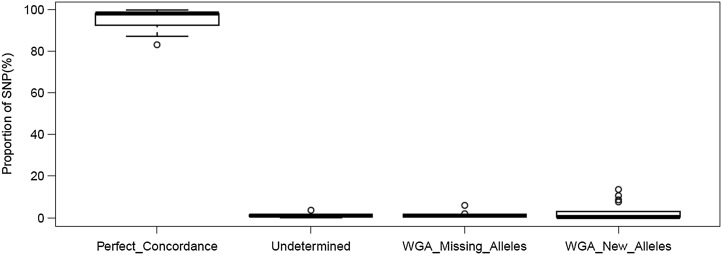
Boxplot of *in silico* genotyping concordance analysing. A total of 20,737 high-quality SNP positions were genotyped in both WGA and non-WGA samples. Call comparison was performed between matched pairs of corresponding WGA and non-WGA datasets and results were grouped into ‘Perfect Concordance’, ‘WGA Missing Alleles’, ‘WGA New Alleles’ and ‘Undetermined’. Perfect_Concordance (identical alleles) had a median of 97.95% (range 83.11–99.58%; *n* = 17). A median of 0.48% calls represent alleles that were called in the WGA samples, but not in the non-WGA samples (WGA_New_Alleles). A median of 0.94% calls were present in non-WGA, but absent in WGA, samples (WGA_Missing_Alleles). The proportion of calls that were missing (Undetermined) had a median of 0.96% (Supplementary Table S4).

Using a list of high-quality SNP positions and alleles from the genetic crosses, we performed *in silico* genotyping of both the WGA and non-WGA samples on 20,737 positions, counting alleles present in at least five reads. Call comparisons between the WGA and non-WGA from same samples were grouped into ‘Perfect Concordance (identical)’, ‘WGA Missing Alleles’, ‘WGA New Alleles’ and ‘Undetermined’ (missing allele). Identical alleles with perfect concordance between WGA and non-WGA samples had a median of 97.95% (Fig. 6). A median of 0.48% calls represent alleles that were called in the WGA, but not in the non-WGA, samples. These reflect cases where the WGA sample had a mixed call and the non-WGA sample had a single-allele call. A median of 0.94% represent alleles that were missing in WGA, but were called in non-WGA samples. Discordant single-allele calls were extremely rare, with a median of zero and a mean of 2.9 SNPs per sample. Four of the 17 samples showed such discordant calls, and only three samples had more than one such call. The 3D7 (3D7_Glasgow) reference sample showed 99.58% identical calls, and only one new allele in WGA sample was returned as a mixed call (Supplementary Table S4).

## 4. Discussion

High-throughput DNA sequencing technologies have gained a wide range of applications in infectious disease research, including global surveillance of the emergence and spread of drug resistance, detection of regions of the genome under selection and identification of genetic determinants of clinical phenotypes through genome-wide association studies. The *P. falciparum* genome presents inherent technical challenges to next-generation DNA sequencing, such as its extreme AT-richness. Whole-genome sequencing of *P. falciparum* field isolates poses additional difficulties due to the small quantity of DNA often retrieved from patients in the clinical setting and the high degree of host DNA contamination from leucocytes. This study addressed the first of these key problems by developing a method for WGA capable of producing high-quality sequence data from just 10 pg of input *P. falciparum* DNA.

Although PCR-based WGA techniques, such as primer extension PCR, ligation-mediated PCR and degenerate oligonucleotide-primed PCR, have been successfully used in some studies such as single-cell amplification,^29^ their wider application has been limited. PCR-based WGA methods produce relatively shorter products, non-specific amplification artefacts and incomplete genome coverage.^19,20,30^ MDA has been associated with base-bias and generation of chimeric products.^29^ Nonetheless, MDA has been the method of choice for a wider range of genome amplification studies, because it produces longer DNA products with extensive genome coverage. MDA also produces higher DNA yields with relatively less amplification bias.^3,11,19^

Here, we have assessed MDA on an AT-rich genome using a range of input DNA quantities. We describe an optimized WGA method incorporating TMAC reagent that improves amplification coverage of the difficult AT-rich loci of the genome. We establish 10 pg of input DNA as the lowest threshold from which our optimized WGA protocol generates an amplification product with optimal *P. falciparum* genome coverage for most genome sequencing analysis. This amount equates to ∼380 parasite genomes, equivalent to ∼1 µl of blood in a patient with 0.01% parasitemia. Furthermore, we show that the optimized conditions significantly reduce the formation of chimeric reads, thereby improving the overall quality of the amplified product.

Standard WGA from such small quantities of input material is often associated with bias and incomplete genome coverage. A single-cell genomic approach that uses infected red blood cell sorting technology has recently been reported that achieved a genome coverage of ∼50% with a standard WGA method.^31^ Although genome coverage in single-cell approach is still low, this technology has opened avenues for single-cell genomics studies in malaria and offers great opportunities for dissecting multiple genotype infection. Our optimized WGA method described here will be useful for optimizing genome coverage in such single-cell genome amplifications, as well as direct field applications for small sample sequencing. For improved malaria clinical sequencing, we routinely employ the combination of host depletion methods^32,33^ and the current optimized WGA procedure to generate high-quality whole genome sequencing data.

We have assessed the quality of the amplified products generated from 10 pg input DNA using high-throughput sequencing of DNA extracted from 17 progeny clones derived from genetic crosses between two laboratory strains. We performed a comparative analysis between the WGA data and their corresponding non-WGA counterparts. We show that coverage was sufficient for allele calling and other whole-genome analyses. Although the number of uncovered bases was slightly higher in WGA (median, 1.5%) than non-WGA (median, 1.2%) samples (Table 1), the difference was insignificant (*t*-test, *P* = 0.053, 95% CI). The same applies to preference for mitochondria DNA amplification, which showed a median of 2.6% in WGA against 0.6% in the non-WGA samples.

Another key aspect of *P. falciparum* whole-genome sequencing from clinical specimens is removing host DNA contamination. This can be achieved either at the blood sample processing stage through leucocyte depletion or through selective enrichment of parasite DNA after extraction.^32–34^ The combination of an effective method for removing human DNA that is applicable to the field setting, and the ability to perform whole-genome sequencing from very low quantities of input DNA as described in this study, has the potential to greatly increase the scope and scale of *P. falciparum* genomic research. This, in turn, would contribute significantly to malaria genetic surveillance and control strategies.

## 5. Conclusion

The optimized amplification conditions described here have generated high-quality whole-genome sequence data (99.8% genome coverage) from a minute amount of input DNA, equivalent to <400 *P. falciparum* genomes. This work shows for the first time that accurate *in silico* genotyping and *de novo* calling of genetic variants is achievable on a WGA sample using <1 ng of input DNA from an extremely AT-rich genome. We anticipate that sequencing from small quantities of input DNA (<1 ng) will become a significant aid to genetic and genomic studies of *P. falciparum* in the field, particularly when combined with effective methods for removal of host DNA contamination.

## Supplementary Data

Supplementary Data are available at www.dnaresearch.oxfordjournals.org.

## Funding

This research was supported by the Wellcome Trust through the Wellcome Trust Sanger Institute (098051), the Resource Centre for Genomic Epidemiology of Malaria (090770/Z/09/Z) and the Wellcome Trust Centre for Human Genetics (090532/Z/09/Z). The Centre for Genomics and Global Health is supported by the Medical Research Council (G0600718). Funding to pay the Open Access publication charges for this article was provided by the Wellcome Trust Sanger Institute.

## Supplementary Material

Supplementary Data
