# Supplementary material for: Optimized Whole-Genome Amplification Strategy for Extremely AT-Biased Template
Source: DNA Res. 2014 Sep 19;21(6):661–71. doi: 10.1093/dnares/dsu028 (PMC4263299; doi:10.1093/dnares/dsu028)
Supplement: Supplementary Data [file supp_21_6_661__index.html]

Optimized Whole-Genome Amplification Strategy for Extremely AT-Biased Template — Supplementary Data 

# Optimized Whole-Genome Amplification Strategy for Extremely AT-Biased Template

## Supplementary Data

Supplementary Data

**Files in this Data Supplement:**

- Supplementary Tables - xls file
